# Supplementary material for: A Novel Zwitterionic Hydrogel Incorporated with Graphene Oxide for Bone Tissue Engineering: Synthesis, Characterization, and Promotion of Osteogenic Differentiation of Bone Mesenchymal Stem Cells
Source: Int J Mol Sci. 2023 Jan 31;24(3):2691. doi: 10.3390/ijms24032691 (PMC9916718; doi:10.3390/ijms24032691)
Supplement: Supplementary file 1 [file ijms-24-02691-s001.zip › ijms-2092407-supplementary.pdf]

Supporting Information

A novel zwitterionic hydrogel incorporated with graphene oxide for bone tissue engineering: Synthesis、characterization and promotion of osteogenic differentiation of bone mesenchymal stem cells

Qidong Wang[a], Meng Li[a], Tianming Cui[b], Rui Wu[c], Fangfang Guo[d], Mei fu[a], Yuqian Zhu[d], Chensong Yang[d], Bingdi Chen[d]*, Guixin Sun[d]*

[a]Department of Traumatic Surgery, Shanghai East Hospital, School of Medicine, Tongji University, Shanghai, China
[b]Shanghai Research Institute for Intelligent Autonomous Systems, Tongji University, Shanghai, China
[c]School of Materials Science and Engineering, Tongji University Shanghai, China,
[d]The Institute for Biomedical Engineering & Nano Science, School of Medicine, Tongji University, Shanghai, China.
* Corresponding authors.
E-mail addresses: inanochen@tongji.edu.cn (Bingdi Chen), sunguixin@sina.com (Guixin Sun).

1. Cell extraction, culture and differentiation of rBMSCs

The Sprague-Dawley (SD) rats used in this research were obtained from t he Animal Experimental Center of Tongji University ((Shanghai, China). The r BMSCs were acquired as described in Reference[1], rBMSCs were isolated and cultured from 4-week-old Sprague-Dawley (SD) rats. All experiments followed the procedure according to the guidelines of the Animal Experimental Ethical Inspection of Tongji University School of Medicine and were approved by the Institutional Animal Care and Use Committee. The femurs and tibias were diss ected, and bone marrow cavities were flushed with Phosphate Buffered Solutio n (PBS). rBMSCs（the bone marrow）were collected, purified by centrifugatio n, and then suspended in a 25 cm^2 flask containing the completed α- Minimu m Essential Medium (α-MEM, Gibco, USA) with 1% penicillin/streptomycin (H yClone, USA) and 10% fetal bovine serum (FBS, Excell, Australia) and placed in an incubator at 37°C with 5% CO2. The culture medium was refreshed ev ery 2 days. The trypsinization and passage were performed when cells reached approximately 80% confluence. Passage 3-4 cells were used for subsequent vitr o experiments. For osteogenic differentiation, cells were influenced by osteogen ic differentiation substrate, as cultured in DMEM supplemented with 50 mg/ml

L-ascorbic acid (Sigma-Aldrich, USA), 1% Pen-Strep, 10% FBS, 100 nM dexa methasone (Sigma-Aldrich, USA), and 10 mM glycerol-2-phosphate (Sigma-Aldr ich, USA)[2]. The osteogenic differentiation medium was replaced every day.

2. Supplementary tables and figures

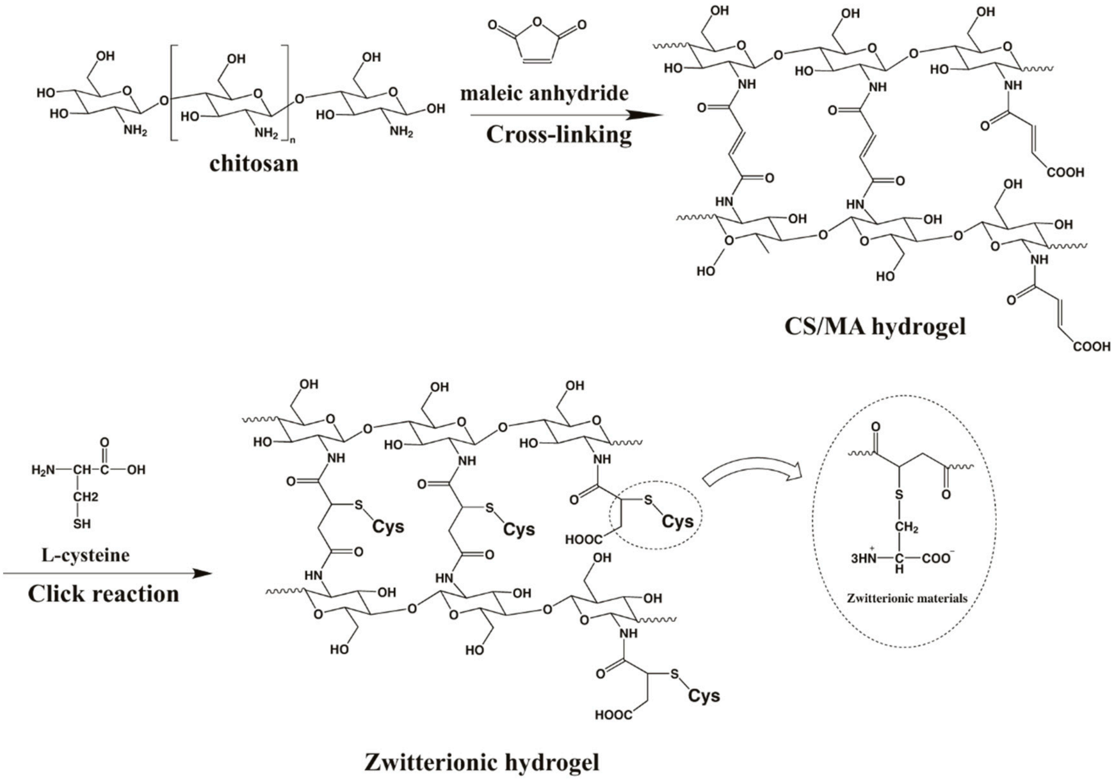

Figure S1. Synthesis of the zwitterionic hydrogel.

Table S1.

Sequences of primers used in the qRT-PCT

Gene	forward and reverse primer sequences
RUNX2	**F:** 5'-CTTCGTCAGCGTCCTATCAGTTCC-3'
	**R:** 5'-TCCATCAGCGTCAACACCATCATTC-3'
OPN	**F:** 5'-GACGATGATGACGACGACGATGAC-3'
	**R:** 5'-GTGTGCTGGCAGTGAAGGACTC-3'
ALP	**F:** 5'-GGCGTCCATGAGCAGAACTACATC-3'
	**R:** 5'-CAGGCACAGTGGTCAAGGTTGG-3'
COL 1	**F:** 5'-TGTTGGTCCTGCTGGCAAGAATG-3'
	**R:** 5'-GTCACCTTGTTCGCCTGTCTCAC-3'
OCN	**F:** 5'-GGACCCTCTCTCTGCTCACTCTG-3'
	**R:** 5'-ACCTTACTGCCCTCCTGCTTGG-3'
β-catenin	**F:** 5'-ACAAGCCACAGGACTACAAGAAACG-3'
	**R:** 5'-TCAGCAGTCTCATTCCAAGCCATTG-3'
GAPDH	**F:** 5'-GACATGCCGCCTGGAGAAAC -3'
	**R:** 5'-AGCCCAGGATGCCCTTTAGT -3'

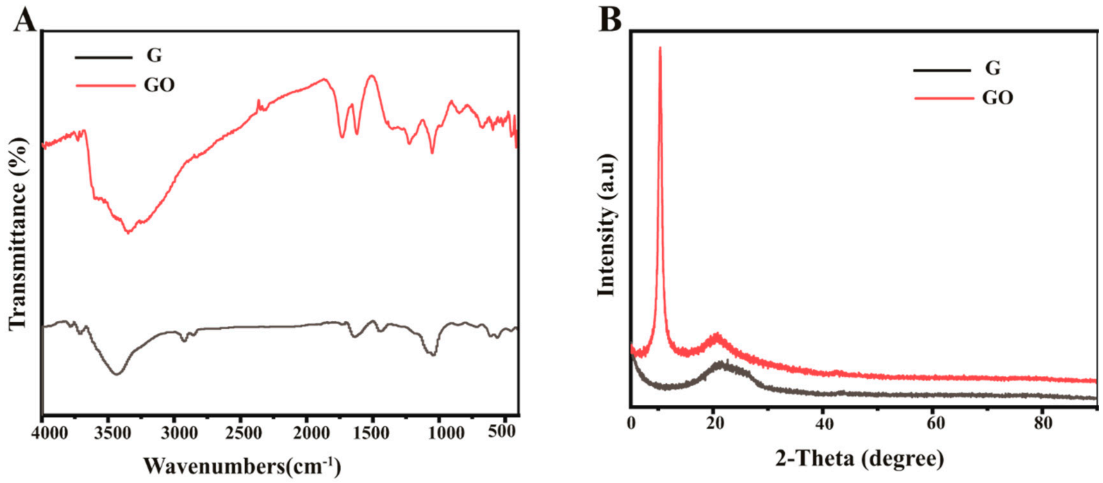

Figure S2. (A) FT-IR analysis and (B) XRD analysis of G and GO

3. surgical procedures

Eighteen male Sprague-Dawley rats of 10 weeks old were randomly divide d into three groups: blank, Z-CS/β-TCP and Z-CS/β-TCP/GO-2 groups. The sur ger y followed these steps,3 after anesthetized by intraperitoneal injection with pentob arbital (4mg/100 g), two symmetrical full-thickness calvarial defects (dia meters of 5 mm) were drilled by dental trephine. The scaffolds were randomly loaded in t he calvarial defect while the group without any scaffolds implante d was the blank group. After implantation, the wounds were sutured by 4-0 sil k sutures. At 6,12 weeks post-surgery, the rats were euthanized by injecting an overdose of pentobarbital.

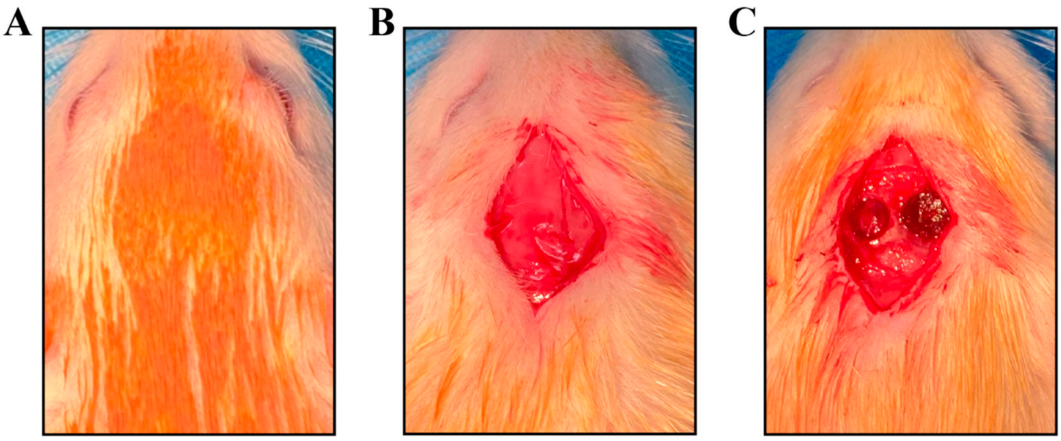

Figure S3. Surgical procedures:(A) disinfection,(B) skin incision,(C) load scaffold (right).

1 Yu, P., Bao, R. Y., Shi, X. J., Yang, W. & Yang, M. B. Self-assembled high-strength hydroxyapatite/graphene oxide/chitosan composite hydrogel for bone tissue engineering.

Carbohydr Polym **155**, 507-515, doi:10.1016/j.carbpol.2016.09.001 (2017).
2 Kim, J. *et al.* Enhanced osteogenic commitment of murine mesenchymal stem cells on graphene oxide substrate. *Biomater Res* **22**, 1, doi:10.1186/s40824-017-0112-8 (2018).
3 Chen, Y. et al. Developing a Strontium-Releasing Graphene Oxide-/Collagen-Based Organic-Inorganic Nanobiocomposite for Large Bone Defect Regeneration via MAPK Signaling Pathway. ACS Appl Mater Interfaces 11, 15986-15997, doi:10.1021/acsami.8b22606 (2019).
